# Precise parallel volumetric comparison of molecular surfaces and electrostatic isopotentials

**DOI:** 10.1186/s13015-020-00168-z

**Published:** 2020-05-25

**Authors:** Georgi D. Georgiev, Kevin F. Dodd, Brian Y. Chen

**Affiliations:** Department of Computer Science and Engineering, Lehigh University, 113 Research Drive, Bethlehem, PA USA

**Keywords:** Specificity annotation, Molecular representations, Solid modeling

## Abstract

Geometric comparisons of binding sites and their electrostatic properties can identify subtle variations that select different binding partners and subtle similarities that accommodate similar partners. Because subtle features are central for explaining how proteins achieve specificity, algorithmic efficiency and geometric precision are central to algorithmic design. To address these concerns, this paper presents pClay, the first algorithm to perform parallel and arbitrarily precise comparisons of molecular surfaces and electrostatic isopotentials as geometric solids. pClay was presented at the 2019 Workshop on Algorithms in Bioinformatics (WABI 2019) and is described in expanded detail here, especially with regard to the comparison of electrostatic isopotentials. Earlier methods have generally used parallelism to enhance computational throughput, pClay is the first algorithm to use parallelism to make arbitrarily high precision comparisons practical. It is also the first method to demonstrate that high precision comparisons of geometric solids can yield more precise structural inferences than algorithms that use existing standards of precision. One advantage of added precision is that statistical models can be trained with more accurate data. Using structural data from an existing method, a model of steric variations between binding cavities can overlook 53% of authentic steric influences on specificity, whereas a model trained with data from pClay overlooks none. Our results also demonstrate the parallel performance of pClay on both workstation CPUs and a 61-core Xeon Phi. While slower on one core, additional processor cores rapidly outpaced single core performance and existing methods. Based on these results, it is clear that pClay has applications in the automatic explanation of binding mechanisms and in the rational design of protein binding preferences.

## Background

Molecular shape and electric fields have a strong influence on binding specificity. At binding interfaces, complementary molecular shapes can accommodate some ligands and hinder those that fit poorly. Electric fields attract molecules with complementing charges and repel others. This connection, between molecular recognition and the complementarity of surfaces and fields, is evidence by which human investigators infer the roles of individual mechanisms in function. Comparison software can make similar inferences. Some methods detect proteins with geometrically conserved binding sites, supporting the inference that they bind similar partners [1–11]. Other methods find variations in electric fields near binding sites, suggesting that they accommodate differently charged ligands [12–15]. These techniques, and their potential for large scale and accurate applications, depend on rapid and precise algorithms for representing and comparing molecular surfaces and electrostatic isopotentials.

This paper presents pClay, an algorithm that uses fine-grained multi-threaded parallelism and mathematically exact representations to achieve more rapid and precise comparisons. In addition to simply making single comparisons faster and more precise, the importance of pClay is that it enhances methods for integrating many comparisons into informed structure-function inferences. For example, binding sites that prefer the same ligand can exhibit many small steric variations. Binding sites that prefer different ligands often have larger variations, because the differences in steric hindrance accommodate different binding partners. Distinguishing small variations between similar binding sites from the bigger variations between different ones can be challenging without some context for what is “large enough”. In such cases, a statistical model, trained on many small variations, can build a frame of reference that can predict atypically large variations that influence specificity [16–19]. The same statistical approach can identify large variations in electrostatic fields that influence specificity [20]. By contextualizing individual comparisons within a framework built from many comparisons, statistical models offer ways to make structure-function inferences that would otherwise rely on human expertise. We hypothesize that statistical models can produce more accurate inferences when trained with data produced with pClay, which rapidly performs more precise comparisons than existing methods.

**Fig. 1:**
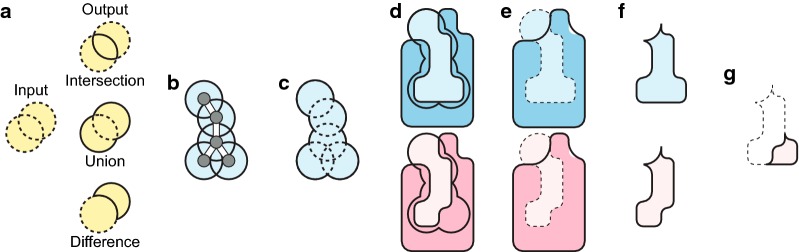
CSG operations on Protein Structure Data. **a** Basic CSG operations. Input solids are yellow with dotted outlines. Output solids have unbroken outlines. **b** Sample ligand with grey atoms and white bonds. Light blue circles are spheres centered on each atom. **c** The CSG union of all spheres in each ligand. **d** The molecular surface of two proteins (blue, red) in complex with each ligand, shown as sphere unions (black outlines). **e** CSG difference of the sphere unions minus molecular surfaces (dotted outlines), shown with molecular surfaces (blue and red, no outline) and envelope surfaces (black outline). **f** Intersection of differences with envelope surfaces (light blue and red, black outlines). **g** The CSG difference between binding cavities reveals a variation in steric hindrance that causes differences in binding preferences

pClay performs comparisons using operations for Constructive Solid Geometry (CSG) (Fig. 1a). These operations, which include unions, intersections and differences, can be combined like arithmetic operators to sculpt geometric solids that represent molecular structure. For example, the union of large spheres centered at ligand atoms can represent the neighborhood of a ligand (Fig. 1b, c). The difference between the spheres and the molecular surface of a receptor can describe the solvent-accessible binding cavity in the receptor (Fig. 1d, e). The CSG difference between one binding cavity and another is the cavity region that is solvent accessible in one protein and inaccessible in the other (Fig. 1g). This sculptural approach inspires both the name pClay, a portmanteau for “protein” and “clay,” and also the solid geometry approach to the analysis of protein shape and charge that pClay enhances. Here, the contribution of pClay is not to introduce CSG-based comparison, which was done earlier (e.g. [13, 21]), but rather to demonstrate that rapid and precise comparison can significantly enhance the speed and accuracy of inferences drawn with CSG-based comparisons.

As input, pClay can accept protein structures as atomic coordinates in three dimensions, which may arise from crystallography or computational molecular models. It can also accept geometric solids, such as spheres, tetrahedra, and the regions within surfaces derived from structural data, including molecular surfaces and electrostatic isopotentials. pClay can be used to output detailed geometric differences between binding cavities, conserved regions of solvent accessibility in binding cavities, regions of electrostatic complementarity, and other structure-function annotations. The regions and surfaces identified by CSG operations are all outputs of pClay, and they make a dual prediction: They predict both the structural influence on specificity (e.g. the red region in Fig. 1g) as well as the steric mechanism by which it acts. Likewise, the CSG-based comparison of electrostatic isopotentials predicts both influential elements of protein structure that and an electrostatic mechanism of action.

**Fig. 2:**
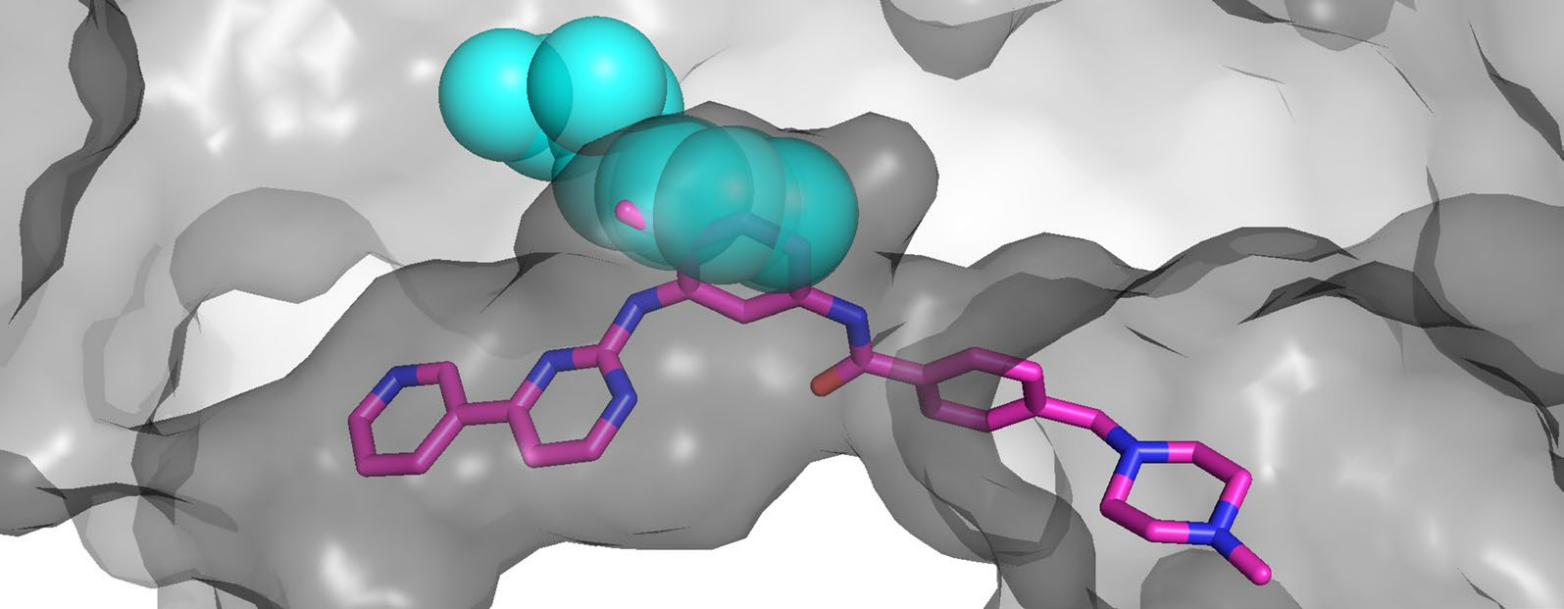
Steric hindrance in the tyrosine kinase active site induced by a large gatekeeper residue. A transparent cross section of the c-Kit tyrosine kinase is shown in gray (pdb: 1t46). The inhibitor imatinib (magenta sticks) is co-crystallized with c-Kit in the active site (dark gray channel). Interleukin-2 tyrosine kinase (pdb: 1snx) is structurally aligned to the c-Kit kinase but not shown for clarity, except for its gatekeeper residue F435 (cyan spheres). This phenylalanine gatekeeper residue is substantially larger than the threonine gatekeeper of c-Kit, creating steric hindrance that prevents longer inhibitors like imatinib from binding in Interleukin-2. Identifying substitutions that influence binding preferences through steric hindrance is one important application of CSG-based comparison methods like pClay

These two-part predictions yield important utility in applications that we have demonstrated earlier. CSG differences between the S1 subsites of the trypsins and elastases can identify threonine 226 which, in elastases, sterically hinders the longer substrates preferred by trypsins that might otherwise bind [21]. That region of hindrance is only 50% larger than a carbon atom ($31\,\AA ^3$), illustrating how important it is to have the precise CSG operations enabled with pClay. A similar approach can identify gatekeeper residues in the tyrosine kinases, which are single amino acids that sterically hinder larger drugs [22]. This application is illustrated in Fig. 2, where a larger phenylalanine gatekeeper can interfere with the binding of imatinib, a larger tyrosine kinase inhibitor.

We have also observed that a CSG-based comparison of electrostatic isopotentials can reveal single amino acids crucial for selecting ligands in the in the cysteine proteases [13] and for stabilizing the three interfaces of the SMAD trimer [14]. In such cases the contribution of a single amino acid to the electrostatic field of the protein is subtle, but the difference is still detected in CSG comparisons of electric fields at the binding site. The correctness of CSG-based predictions was further demonstrated when a blind prediction of the electrostatic importance of arginine 235 was later verified experimentally on a study of the ricin toxin [15]. Since no large databases currently link individual mutations to biophysical mechanisms of action, a larger scale validation of the CSG-based approach is not possible. To these smaller-scale studies, however, pClay contributes parallelism, for enhanced throughput, and precision, up to machine limits, to ensure that subtle but influential details are not overlooked.

The precision that pClay achieves derives from geometric solids that have analytical representations. pClay can assemble these primitives into solvent excluded regions, which we call molecular solids. The boundary of a molecular solid is the classic molecular surface, also known as the solvent excluded surface or Connolly surface, which was originally developed by Richardson and others [23, 24]. While we can construct molecular solids with CSG operations on many individual primitives, pClay exploits molecular properties to sidestep those operations and achieve greater efficiency. The resulting molecular solids avoid the “photocopier effect,” where multiple CSG operations can accumulate geometric errors. They can also be translated into triangle meshes at an arbitrary degree of precision.

pClay also performs CSG operations on any closed triangle mesh as if it was an exact geometric solid. Much like VASP-E, this capability enables CSG operations to be performed on electrostatic isopotentials, such as those produced by GRASP2 [25]. In such cases, the electrostatic isopotential, a surface, is used to define the boundary of an electrostatic solid. Since electrostatic solids and molecular solids are simply geometric solids with different origins, pClay can express and compute CSG operations that combine electrostatic isopotentials and molecular surfaces. These data can be used to identify electrostatic influences on specificity based on their locality to binding cavities and their complementarity with other charges, as we have shown in the past [13–15]. Also, because triangle meshes are treated as exact solids, electrostatic isopotentials never lose precision after their original approximation into meshes.

pClay boosts computational efficiency with parallelism. As a result, CSG expressions can be evaluated more rapidly than they would have been on a single processor core. We achieve parallelism in pClay in a number of ways, most notably by recasting Marching Cubes, a traditional method for implementing CSG operations [26, 27], into a series of parallel breadth first searches (BFS). In pClay, we use BFS to traverse cubic lattices and identify contiguous regions of cubes within defined boundary regions. These breadth first traversals can be distributed evenly across arbitrary numbers of threads. By dividing the computation in this way, parallelism can make comparisons faster and also enable more detail to be considered. This advancement stands in qualitative contrast with existing efforts to parallelize structure comparisons (e.g. [4]), where throughput was increased without benefiting precision. To demonstrate the parallel scalability of our method, pClay was tested on both modern multicore processors as well as on a Xeon Phi, a manycore coprocessor with 61 cores.

In our experimental results, pClay achieved precise, scalable performance on range of problem sets. First, we demonstrate that pClay generates molecular solids that are essentially identical to those generated by existing algorithms. Second, we show that pClay exhibits substantial parallel speedup on a range of CSG operations representing both realistic and artificial applications. Finally, we demonstrate an example application of pClay, where a statistical model trained on data from pClay has substantially improved prediction accuracy over the same model trained with data from earlier methods. These results point to a range of applications in automatically inferring the functional role of steric and electrostatic elements of protein structure in molecular recognition.

## Related work

VASP [21] and VASP-E [13] were the first algorithms to use CSG-based comparison to identify elements of protein structures that influence specificity and connect them to steric and electrostatic mechanisms of action. pClay advances on these methods by enabling representations of molecular structures that are exact up to machine precision and by performing CSG operations in fine-grained parallelism. By distributing comparisons over over multiple processors, pClay enables comparisons to be performed at larger scales and at degrees of precision that were previously impractical. While pClay is the first algorithm to integrate arbitrary precision and parallelism to perform comparisons of molecular structure, aspects of these capabilities exist separately in methods for other applications.

One such application is molecular visualization, which has often made use of high precision molecular surfaces for visual clarity. Molecular surfaces, also known as solvent excluded surfaces or Connolly surfaces, are commonly generated as a collection of points [24, 28], arcs [23] or as triangle meshes [25, 29–32] in visualization applications (e.g. [33, 34]) and for calculating solvent accessible surface area (e.g. [35]). For these applications, existing methods could be modified to generate molecular surfaces at arbitrary degrees of precision, but most operate at a fixed precision because more detail is unnecessary: Meshes that are finer than those needed for visualization are more difficult to render on the same hardware, they take longer to generate, and the added refinement may not be visible to a user. They also yield biologically insignificant refinements to calculated surface area. While the design of pClay has similarities to these methods, especially in that it is also inspired by the Shrake-Rupley approach [28], it uses arbitrary precision representations to support comparison rather than visualization. In the comparison scenario, arbitrarily fine resolutions yield more precise comparisons and more accurate predictions, as we shall demonstrate in our results.

Several recent methods do generate arbitrarily precise representations of the molecular surface. Techniques using NURBs [36], alpha shapes [37] or spherical coordinates [38, 39] fall into this second category. Generally, these techniques for surface generation have been used for visualization and not for computing CSG operations, though NURBs and perhaps others are compatible with CSG applications. In this regard, pClay explores new applications of CSG on arbitrarily precise representations of molecular surfaces. pClay also differs from existing methods because it generates molecular solids from collections of three dimensional solids rather than by connecting surface patches or generating surface approximations.

The comparison of protein structure is frequently parallelized because large scale comparisons can be used to build statistical models and scan for remote homologs. MASH [4] was the first such parallel comparison algorithm and it demonstrated that parallel distributed protein structure comparison could refine motifs as geometric search terms for remote homologs, and it achieves a superlinear speedup in doing so [4]. Parallel-Probis achieves parallel speedups for the direct parallelization of large database searches [40]. More recent methods use cloud based resources to make parallel structure search more accessible to other users [41]. In contrast to these efforts to perform more comparisons more quickly, pClay uses parallelization different to add higher precision as well as faster performance.

For applications in computer assisted design, algorithms for visualizing the output of CSG operations have been parallelized to create efficient user interfaces (e.g. [42]). These efforts do not generate an explicit surface as pClay does, nor do they yield analyzable volume data. CSG operations may be decomposed into components and processed in parallel (e.g. [43] ), for additional performance.

## Methods

As input, pClay accepts a collection of geometric solids and an expression of CSG operations. We convert the CSG expression into a binary tree, a CSG tree, where the nodes of the tree are geometric solids. The input solids, which include spheres, spindles, tetrahedra, molecular surfaces or triangle meshes, are leaves on the CSG tree, while the result of CSG operations are the non-leaf nodes. The final result of all operations, the root node, is the output. pClay can also generate a closed triangular mesh at user-defined resolutions to approximate the boundary of the output.

To perform CSG operations, pClay implements a parallel version of Marching Cubes [26] (the “Parallel marching cubes” section), which we summarize below. Our method requires three basic functions to be performed by every geometric solid. These functions are containsPoint(), intersectSegment(), and findSurfaceCubes(). Given any point p in three dimensions, containsPoint(p) determines exactly if p is inside or outside the solid. A point exactly on the surface is said to be inside the solid. Second, given a line segment s, intersectSegment(s) determines all points of intersection between the surface of the operand and s, as well as the interior or exterior state of each interval on the segment. Finally, given a cubic lattice l that surrounds the primitive, findStartingCubes(l) finds a few cubes of the lattice that are *surface cubes*, having at least one corner inside and one corner outside the solid. These cubes are used to initiate a parallel breadth first search for all surface cubes, called findAllSurfaceCubes(), which is implemented once for all primitives and described in the  “Finding all surface cubes” section. To implement each leaf node it is thus sufficient to describe how these basic functions are implemented for that solid. Non-leaf nodes implement the basic functions as logical operations, as we will detail later.

Below, we first describe how the output approximations are generated using a parallelization of Marching Cubes and how we find all surface cubes beginning the output from the starting cubes generated by the basic function. We next explain how the three basic functions are implemented for every primitive. Finally, we detail how the basic functions are implemented in non-leaf nodes.

### Parallel marching cubes

**Fig. 3:**
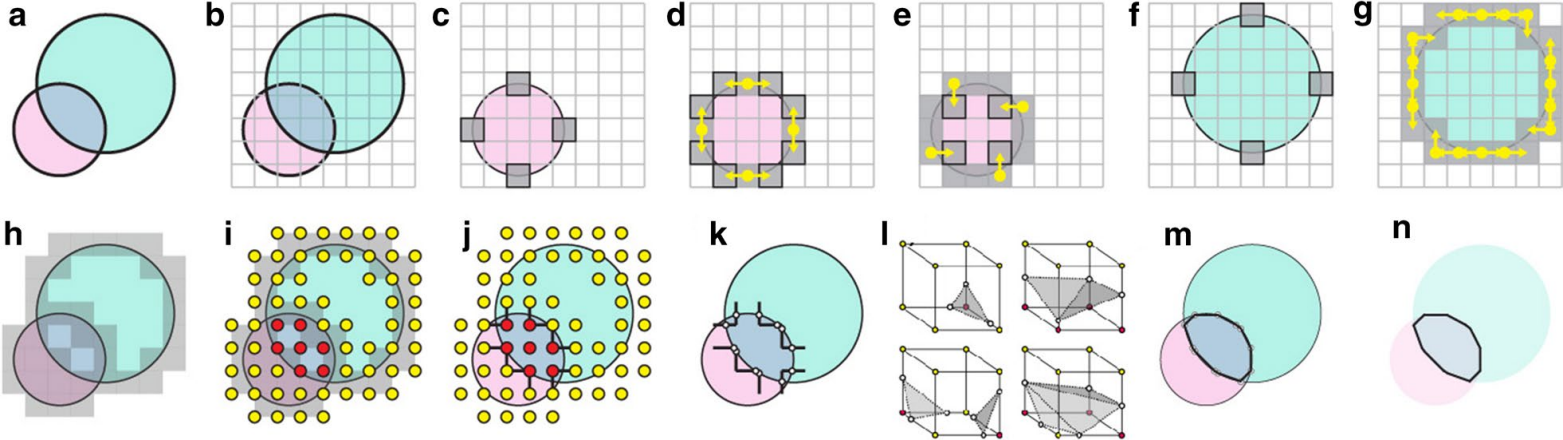
**a** Input operands, shown in red and green solids, with black outlines. **b** Cubic lattice surrounding operands (gray). **c**, **f** Surface cubes for both operands (gray boxes). **d**, **e**, **g** Several steps of floodfill propagation (starting at yellow circle, following yellow arrow). **i** Corner points of each surface cube (joined gray squares) tested for exterior (yellow) or interior (red) state. **j** Segments that cross the boundary of the output surfaces (Black lines). **k** Intersection points (white circles) where the segments intersect the output surface. **l** lookup table representing three dimensional surface constructions with different edge intersection patterns. **m** Triangles (black lines) approximating the intersection points (gray). **n** Final output surface, black lines

As input, Marching Cubes accepts a set of geometric solids (Fig. 3a), which we will refer to as operands, and a CSG expression tree to be performed on the operands. It also accepts a resolution parameter in angstrom units that specifies the degree to which the result of the CSG expression should be approximated in the output.

We begin by defining l, an axis aligned cubic lattice surrounding the input operands, where each cube has sides equal to the user-specified resolution parameter (Fig. 3b). This step is performed by examining the sizes of all operands and the related CSG operations.

Once the lattice is defined, we invoke findStartingCubes(l) on each input solid (Fig. 3c, f). The surface cubes identified are provided as input to findAllSurfaceCubes(), which identifies all remaining surface cubes of all inputs solids in parallel (Fig. 3h). The process of identifying surface cubes for all input solids also necessarily determines the interior/exterior state of the points on these cubes in relation to specific solids. We then compute the interior/exterior state of these points in relation to all other solids in an embarrassingly parallel manner. Once this assessment is made for any point, we can access whether that point is inside or outside the output region (Fig. 3i). In this way, we find the subset of cubes that contain a corner inside and a corner outside the output region.

Next, on each cube of the output surface, we identify edges that connect one corner that is inside the output region to one that is outside (Fig. 3j). Since these edges must pass through the output surface, we call segIntersect() on the root node to find the point of intersection between the edge and the output surface (Fig. 3k). This process is parallelized across the list of edges, ensuring that the calculation is never duplicated when dealing with adjacent cubes.

Finally, once intersections for every edge on every surface cube are determined, triangles are generated in each cube following a lookup table (Fig. 3l). The collection of all resulting triangles form a closed triangular mesh that approximates the output region (Fig. 3m, n).

### Finding all surface cubes

findAllSurfaceCubes() accepts a cubic lattice l (Fig. 3b), a list of starting cubes c (e.g. Fig. 3c,f), and a primitive p for which to find all remaining surface cubes. We perform a parallel floodfill algorithm to identify the remaining surface cubes: Each available thread is assigned a surface cube. Each thread then tests cubes adjacent to the assigned cube to find any that are also on the surface of the input solid (e.g. Fig. 3d). This test is performed by calling containsPoint() on the corners of the adjacent cube. If at least one corner is inside the input solid and another corner is outside, the adjacent cube is stored on a queue of upcoming cubes. Once all cubes adjacent to the initial surface cubes have been either added to the queue or discarded, all threads are then directed to find cubes adjacent to those still on the queue (e.g. Fig. 3e), and so on, until the queue is empty, and all cubes on the surface of the input solid have been identified. Duplicate entries onto the queue are eliminated by recording previously-examined cubes on a hash table.

### Input solids (leaf nodes)

pClay supports several kinds of simple and complex solids for CSG operations. These are spheres, tetrahedra, spindles, molecular surfaces and polyhedral meshes. Each solid type must support the three basic functions: containsPoint(), intersectSegment(), and findSurfaceCubes(). Thus, to describe the implementation of these solids, we describe how each method is implemented for the solid.

#### Spheres

**Fig. 4:**
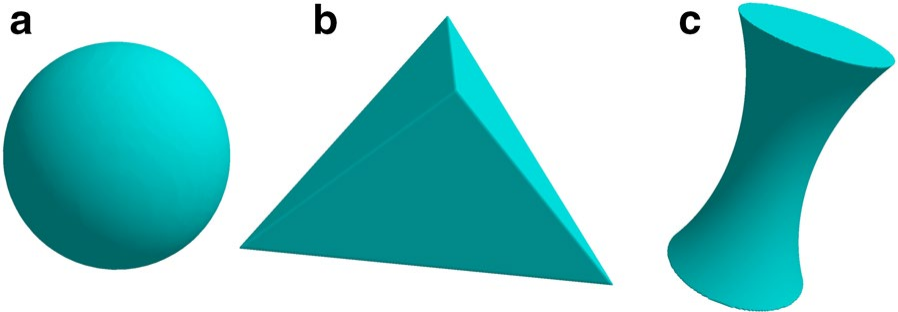
**a** A sphere. **b** A tetrahedron. **c** A spindle

Spheres (Fig. 4a) are defined by center point and radius. containsPoint(p) is implemented by determining if the distance from a point p to the center point of the sphere is at most equal to the radius. To compute intersectSegment(s), we recast the problem on the plane coplanar with the segment and the center of the sphere, where it reduces to the trivial problem of finding intersections between a line and a circle. In the rare case where the segment intersects the sphere exactly at a point of tangency, two artificial points of intersection are generated at a trivial separation to maintain topological consistency. findStartingCubes() is implemented by identifying the cube that contains the center of the sphere. From this center cube, we step outwards, along adjacent squares, in the six orthogonal directions until we find six cubes that are partially inside and partially outside the sphere (e.g. Fig. 3c, f).

#### Tetrahedra

Tetrahedra (Fig. 4b) are defined by four points in space. containsPoint(p) is implemented by determining if the point p exists on the correct side of the four half-planes that define the faces of the tetrahedron. intersectSegment(s) is implemented by identifying points of intersection between a given segment and each triangle face of the tetrahedron. In the rare case where the segment intersects the tetrahedron at a point of tangency to an edge or to a corner, two artificial points of intersection are generated at a trivial separation to maintain topological consistency. Where the segment is colinear with the edge or face of the tetrahedron, the interval returned is the interval of overlap. Finally, findStartingCubes() is implemented by first identifying the cubes that contain each corner of the tetrahedron. In some cases, these cubes do not have both a corner that is inside and a corner that is outside the tetrahedron. In that case, we generate the vector from the tetrahedron corner to the center of the opposite face of the tetrahedron, we find the face of the corner cube that this vector passes through, and identify the cube on the other side. We then repeat our check for interior and exterior corners on that cube, repeating again as necessary until we reach the center of the opposite face. We repeat this process for each of the four corner points, and if no surface cube is identified, none are returned.

#### Spindles

**Fig. 5:**
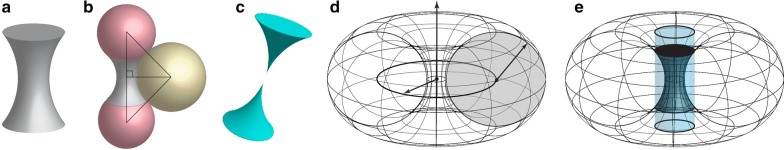
**a** Spindle. **b** Formation of a spindle (gray) in a simple molecular surface defined on two atoms (red) and a solvent sphere (yellow). The perpendicular projection of the center of the solvent sphere onto the interatomic axis defines the center point. **c** “broken” spindle. **d** Torus defining some characteristics of a spindle, including center point (black dot, center), perpendicular vector (vertical arrow), major radius (arrow from center point to horizontal ellipse), minor radius (diagonal arrow from horizontal ellipse to torus surface. **e** Cylinder (light blue with black outline)

Spindles (Fig. 5a) define the solvent excluded region between two atoms that are too close to permit a sphere representing a solvent molecule to pass between them (Fig. 5b). “Broken” spindles (Fig. 5c) occur when the edge of the solvent sphere can pass beyond the centerline of the two atoms. Conceptually, spindles are the volume within a cylinder minus the volume within a coaxial torus. We define spindles by center point, perpendicular vector, major radius, and minor radius taken from the torus (Fig. 5d), and end cap positions along the perpendicular vector (fig. 5e). The center point is the perpendicular projection of the center of the solvent sphere onto the segment between atom centers. The perpendicular vector points from the center point towards the center of one atom. The major radius is the radius of the circle defined by the center of the solvent sphere as it rotates about the two atoms. The minor radius is the radius of the solvent sphere. The endcaps are circles perpendicular to the perpendicular vector that are defined by the point of tangency between the solvent sphere and the atoms, as the solvent sphere rotates about the atoms. The boundary surface of a spindle is defined by the end caps and elsewhere by the interior curve of the torus (Fig. 5d).

To implement containsPoint(*p*), note that the spindle is rotationally symmetric about the perpendicular vector. Thus, a plane *K* can be defined coplanar to *p* and the perpendicular vector of the torus. In *K*, *p* is inside the spindle only if it is inside the rectangle that defines the rotational cross section of the cylinder and also outside the circle that defines the rotational cross section of the torus.

intersectSegment(*s*) is computed by first setting up the calculation by translating the center of the spindle to the origin and rotating its axis to align it with the x axis. The segment *s* is translated and rotated with it. We can describe the torus aligned to the x axis as follows:

1 $$\begin{aligned} (x^2+y^2+z^2 + R^2 - r^2)^2 - 4R^2(y^2+z^2) = 0 \end{aligned}$$

where *R* is the major radius, and *r* is the minor radius of the torus. In the torus equation, we substitute *x*, *y* and *z* with the line expressions $x_0+td_x, y_0+td_y, and z_0+td_z$, where $x_0, y_0, z_0$ are segment starting points, and *t* parameterizes the line containing the line segment. The result of this substitution is a quartic equation on *t*, and roots of the equation will be parameters on the segment at points of intersection between the segment and the torus. We converted this equation into a monic quartic using Maxima, a computer algebra system [44].

To find the solutions of this equation, we produce the Frobenius companion matrix of this quartic polynomial. The roots of Eq. 1 are the eigenvalues of this matrix. Complex eigenvalues correspond to nonexistent points of intersection between the segment and the torus, while real eigenvalues correspond to intersection points on the torus. We find these intersection points and eliminate any intersections that are outside of the cylinder. Separately, we also find intersections with the end caps of the spindle, treating them first as infinite planes and then determining if the intersection point is within the circle on the plane. Intersections between the segment and the endcaps or between the segment and the torus are returned as intervals where the segment is inside the spindle.

findStartingCubes() is implemented by first generating the segment between the centers of the endcaps. The lattice cube containing one centroid is identified, and if it is not a surface cube, the adjoining cube, through whose face which the segment passes, is identified as the next cube to examine. This process is repeated until either the segment ends at the other centroid of a surface cube has been found. In the case where the spindle is broken (Fig. 5d), two segments are generated, starting at one endcap centroid and moving towards the other endcap centroid, but ending at the center.

#### Molecular solids

pClay generates molecular solids by positioning structural components with the power diagram [45]. This approach follows the classic methods for generating molecular surfaces, such as CASTp [37], MSMS [29], GRASP2 [25], which also use power diagrams or similar constructs. For this reason, we paraphrase our approach here, expanding on points that differ from classic methods. As in the earlier methods, our approach represents water molecules as solvent spheres, which can be of any given radius. By calling basic functions from simpler primitives, pClay achieves an efficient implementation of the basic functions for the entire molecular solid without describing it as a CSG operation of many individual primitives.

**Fig. 6:**
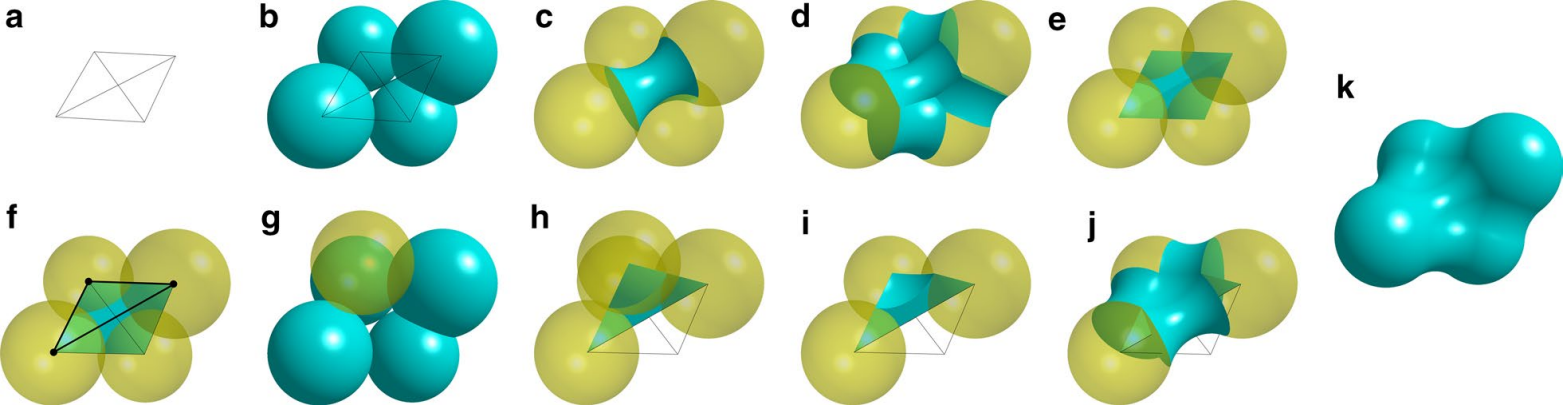
Molecular Surface Construction. **a** Dual graph of a power diagram on four atoms (graph edges shown with black lines, graph vertices shown as corners). **b** Sphere primitives from atoms (teal) shown with dual graph. **c** Atoms (transparent yellow) with one spindle (teal). **d** Atoms with spindles corresponding to all edges of the dual graph. **e** Tetrahedron primitive (teal) with atoms (yellow). **f** One triangle of the dual graph (bold lines, black circles) that is not between tetrahedra. **g** Solvent sphere (yellow) tangent to three of the atoms (teal). **h** New tetrahedron (teal) with corners in the center of the three atoms of the triangle and the solvent sphere (yellow). **i** Cup region inside the new tetrahedron and outside the solvent sphere (teal) shown with three atoms of the triangle (yellow). **j** Cup, shown with three adjacent spindles (teal) and three atoms of the triangle (yellow). **k** Finished molecular solid

We begin with an input file from the Protein Data Bank (PDB). Using atomic coordinates and Van der Waals radii for each atom, we first compute a power diagram with REGTET [46]. The power diagram divides three dimensional space into cells corresponding to each atom of the input. The size of a cell relates to the Van der Waals radius of the atom, through the power function. Using the power diagram, we construct a topologically dual geometric graph (Fig. 6a), which has a vertex at the center of each atom and an edge between any vertices that correspond to adjacent cells. This dual graph defines the location of the primitives that will comprise the molecular solid. In sequential stages, we generate all primitives of the same type in parallel, starting with sphere primitives, then spindles, tetrahedra, and so on.

At every vertex of the dual graph, we create sphere primitives with the appropriate Van der Waals radius of each atom (Fig. 6b). Next, we examine every edge on the dual graph and generate a spindle between the atoms on at the endpoint of each edge, except for overlong edges that are longer than the sum of Van set Waals radius of the endpoint atoms and the diameter of the solvent sphere (Fig. 6c, d). Once all spindles are completed, we identify all tetrahedra in the dual graph that lack an overlong edge and we generate a tetrahedron primitive for each one (Fig. 6e).

Next, we identify triangles on the dual graph that are not between two tetrahedra (Fig. 6f). These triangles define triplets of atoms that may be on the molecular surface. To determine whether the atoms are on the surface, we place a solvent sphere tangent to all three atoms (Fig. 6g). If the solvent sphere does not collide with any other atoms, we create a negsphere: a sphere primitive in the tangent location in the same size as the solvent that describes a region of the solvent outside the molecular surface. We also generate a tetrahedron with corners on the triangle and at the center of the negsphere (Fig. 6h). The region inside this tetrahedron and outside the negsphere is both inside the solvent excluded region and not occupied by spindles or atoms or other tetrahedra. We call this concave subset of a tetrahedron a cup (Fig. 6i), and describe cups as a negsphere-tetrahedron pair. The concave surface of the cup is continuous with the three adjacent spindles and atoms (Fig. 6j). Once all triangles that are not between two tetrahedra have been examined for the presence of a cup, the combination of spheres, spindles, tetrahedra and negspheres form a molecular solid (Fig. 6k).

To support the three basic functions, we store all of these primitives in a data structure for rapid range-based lookup. First, we generate a bounding box for each primitive. Next, we generate a lattice of cubes, where each cube is 2 angstroms on a side. Finally, we associate each primitive with all lattice cubes that intersect its bounding box. These associations act as a hashing function that enables us to rapidly identify any primitives nearby a given cube in the lattice. Since real molecules have finite atomic density, and since primitives are constructed from atoms and between atoms, the number of primitives associated with any cube is finite. As a result, a hashing function based on the lattice achieves algorithmically constant time lookup of nearby primitives.

A) containsPoint(p) Given a point p, if p is outside the coarse lattice, then we immediately return false, because p must be outside the molecular surface. If not, we determine which cube c of the coarse lattice contains p. Next, we identify all primitives associated with c. We use the containsPoint() function of each associated primitive to determine if p is inside the primitive. If p is inside a negSphere, then p is outside the molecular surface. if p is inside any other primitives, then p is considered inside the molecular surface. If p is not inside any primitives, it is outside.

B) intersectSegment(s) Given a segment s, we generate a list of cubes C that contain some interval of s. Next, we generate a list of primitives P associated with the cubes in C. We then query each primitive p in the list P for an interval of intersection between p and s using the intersectSegment() method of each primitive. The output intervals generated are the union of the intervals in tetrahedra, spindles and spheres minus the union of intervals inside negspheres.

C) findStartingCubes(l) During the construction of the molecular solid, we record the points of tangency between all negspheres and atom spheres. For each of these points, we identify the lattice cubes of l that contain them. We also generate starting cubes from all spindles and isolated spheres in the protein structure, calling findStartingCubes() on each of these primitives. From these cubes, we return only cubes that exhibit one corner inside and one outside the molecular solid.

#### Polyhedral meshes for electrostatic analysis

pClay performs CSG-based comparisons of electrostatic isopotentials by representing them as polyhedral meshes that are interpreted as geometric solids. Beginning with a pdb file, we provide the atomic coordinates to DelPhi [47], a widely used program for producing finite difference solutions to the Poisson-Boltzmann equation. DelPhi produces an electrostatic potential field that estimates the electrostatic potential at points within and surrounding the provided structure. Next, we use VASP-E to analyze the field and generate an isopotential surface at a given threshold *k*. When *k* is positive, the surface surrounds positively charged with electrostatic potential equal to or larger than *k*. When *k* is negative, the surface describes negatively charged regions with potential equal to or less than *k*. When evaluating the electrostatic complementarity of two interacting molecules, we evaluate the CSG intersection of a positive isopotential from one molecule and the negative isopotential from the other molecule, and vice versa. As we showed in earlier work [13], amino acid substitutions that cause large changes in complementarity can identify residues that have a strong electrostatic role in binding specificity.

**Fig. 7:**
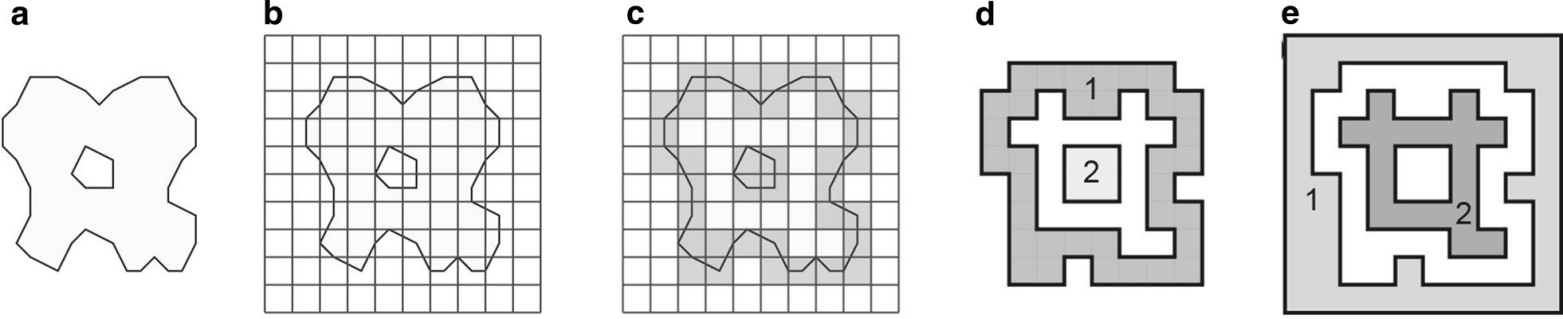
Processing Polyhedral Meshes. **a** Polyhedral mesh (light green), with boundary triangles drawn as black segments. An internal void (white, center) with boundary line segments. **b** A cubic lattice surrounding the mesh. **c** Non-empty cubes (gray squares) and empty cubes (transparent squares). **d** Two distinct connected components of non-empty cubes (numbered). Two distinct connected components of empty cubes (numbered)

The surface generated by VASP-E is a closed polyhedral surface composed of triangles, which pClay interprets as a geometric solid (Fig. 7a). To be able to perform CSG operations on mesh primitives, we prepare it by, first, constructing a cubic lattice surrounding the mesh with cube size equal to the resolution (Fig. 7b). Second, in parallel, each triangle of the mesh is associated with any cube in the lattice it passes through, generating a hash table for looking up triangles in a given cube. In this process, all cubes in the lattice are classified as empty or non-empty: Cubes containing triangles are called non-empty, and the remaining cubes are empty (Fig. 7c). Third, using a parallel breadth first search, the non-empty cubes are categorized into connected components, where non-empty cubes that share a face are considered part of the same connected component (Fig. 7d). Through parallel breadth first search, we also categorize empty cubes into connected components (Fig. 7e).

Next, we determine whether connected components of empty cubes lie nested within each other, based on adjacent non-empty cubes. This assessment is computed from the outside inwards: The cubes at the edge of the lattice are exterior by definition; the empty cubes on the other side of the first set of nonempty cubes are always interior, and so on. We apply this alternating assignment process to each group of nested empty cubes, checking for topological abnormalities. An abnormality may arise if a given triangle mesh has an internal void with a boundary so close to the surface that they exist within a single lattice cube, then empty cubes of the void could be incorrectly assigned. We check for these cases by generating a line segment between sets of empty cubes and counting the number of intersections that occur with triangles in the mesh. An odd number of intersections implies that the two sets of empty cubes have opposite interior/exterior status, while an even number implies that they are the same. The result is a categorization of all connected components of empty cubes as either “empty-interior” or “empty-exterior”.

**ContainsPt(p)** Beginning with an input point p, we first find the lattice cube that contains it. If p is outside the lattice, or inside an empty-exterior cube, then containsPt() returns false. If p is in an empty-interior cube, then true is returned. Finally, if p is inside a non-empty cube, 5 line segments are randomly generated between p and the centers of nearby empty cubes. We then count the intersections between these line segments and triangles of the input mesh. For segments connecting to empty-interior cubes, an even number of intersections votes that p is interior; an odd number of intersections votes that p is exterior. Segments connecting to empty-exterior cubes generate opposite votes for interior and exterior status. Once all segments are examined, votes are counted, and the majority is used to determine if p is interior or exterior.

**intersectSegment(s) ** Beginning with a segment s as input, we first determine all lattice cubes that intersect the segment, and then retrieve all triangles associated with these cubes. Next, we find all the intersections between s and the triangles. Using containsPt(), we determine the interior/exterior status of the first endpoint of the segment, then use it’s status to determine the interior/exterior status of the intervals between intersections along s.

**findStartingCubes(l) ** Given an input lattice l that is distinct from the lattice m constructed for the Polyhedral Mesh primitive, we must identify starting cubes in l. We perform this process by selecting cubes c from the nonempty cubes in m, making sure that every connected component of nonempty cubes yields at least one cube in c. Taking the cubes in c, we find cubes in l that overlap with those in c, and then use containsPt() to determine if they have corners both inside and outside the mesh. The cubes with corners inside and outside the mesh are returned as starting cubes.

#### CSG operations: union, intersection, and difference

A CSG operation node represents the outcome of a CSG operation on its operand nodes. Thus, it is responsible for fulfilling the three basic functions as if it was a primitive, and it implements those functions by calling on its operand nodes. We refer to the operand nodes in the text below as *A* and *B*.

**ContainsPt(p) ** For a given point p, the CSG Union returns true if containsPoint(p) returns true on at least one operand, and false otherwise. The CSG Intersection returns true if containsPoint(p) returns true on both operands, and false otherwise. The CSG difference between operands *A* and *B* returns true if *A*.containsPoint(p) is true and *B*.containsPoint(p) is false, and false otherwise.

**intersectSegment(s)** For a given segment s, intersectSegment(s) on any CSG operation begins by separately calling intersectSegment(s) on operands *A* and *B*, which separately output intervals *a* and *b*. The output of intersectSegment(s) on a CSG Union is the union of *a* and *b*, the output on a CSG Intersection is the intersection of *a* and *b*, and the output on a CSG difference is the subset of the *a* that is not in *b*.

**getSurfaceCubes() ** Given a cubic lattice *l*, calling getSurfaceCubes() on a CSG union, intersection, or difference returns the setwise union of cubes returned by calling *A*.getSurfaceCubes() and *B*.getSurfaceCubes(). We always return a union of cubes because examining the union of cubes can avoid circumstances where a disconnected region in the final solid is lost. Performance profiling reveals that considering the union of all cubes is a minor aspect of overall performance, except in the case of CSG operations that are artificially constructed to create many irrelevant cubes.

### Evaluating the correctness of molecular surfaces

We evaluate the accuracy of molecular surfaces produced with pClay by measuring their similarity to surfaces produced by the trollbase library, an established tool for molecular surface generation in GRASP2 [25], VASP-E [13], and MarkUs [48]. Surfaces generated with the trollbase library have fixed resolution, while surfaces generated with pClay were created at 0.25 Å resolution. pClay is capable of substantially finer resolutions, but 0.25 Å was chosen to create surfaces with similar numbers of triangles. Evaluating whether the surfaces generated are similar in many places is a more stringent test because the number of triangles is limited.

**Fig. 8:**
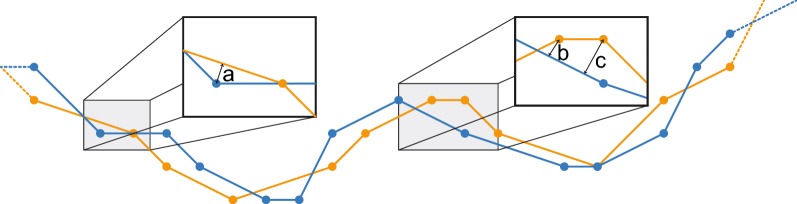
Computing displacement distances. Two similar but nonidentical polyhedral meshes are shown as orange and blue line segments ending in circles. Line segments represent individual triangles, and circles represent triangle corners. At every point on both meshes, the displacement distance is the minimum distance to the other mesh, as shown by thin lines with arrowheads at both ends (a, b, c)

First, we compare the volume contained by the surfaces using the Surveyor’s Formula [49]. To paraphrase this method, the Surveyor’s formula divides any nonconvex solid into positive and negative tetrahedra and computes the total volume from those tetrahedra. Since comparisons of total volume do not strictly prove shape similarity, we also measure the distance between every corner of triangle meshes produced by pClay and the nearest point on the surface produced with trollbase (Fig. 8). This distance is measured even if the nearest point is in the middle of a triangle, or on the edge or corner of a triangle, and we call it the *displacement distance*. To evaluate shape similarity, we compute minimum, maximum and average displacement distances over whole surfaces.

### Comparison of pClay with VASP

To our knowledge, VASP [13] is the only other algorithm for comparing molecular and electrostatic solids. VASP makes these comparisons by performing CSG operations on polyhedral solids, without exact primitives or parallelism. Nonetheless, VASP has demonstrated an ability to identify important steric [16, 21, 50] and electrostatic [13–15, 51] components of protein structure that control specificity. For this reason, we compare both the performance and the accuracy of pClay to VASP.

### Solid representations of binding cavities

We describe binding cavities as geometric solids using CSG operations (Fig. 1). We begin with a collection of aligned protein structures and a ligand bound to one structure (Fig. 1b). First, we compute the union of 5.0 Å spheres centered on the ligand atoms (Fig. 1c), to describe the neighborhood of the ligand. Next, we generate a molecular solid from the protein structures with a 1.4 Å radius probe sphere (Fig. 1d) to represent the region that is inaccessible to solvent. A second molecular solid is generated with a 5.0 Å radius probe sphere to represent the region that is inaccessible to molecular fragments larger than 10 Å in diameter (Fig. 1e). This *envelope solid*, developed originally for SCREEN [52], defines the exterior boundary of the cavity. The CSG difference of the sphere union minus the molecular solid (Fig. 1e, dotted outlines), intersected with the envelope solid (Fig. 1f), produces the solid representation of the binding cavity.

### Implementation details

pClay is implemented in C and C++. A C wrapper supports REGTET [46], a fortran program for computing power diagrams. Parallel communication and coordination was achieved in part with Intel’s Threading Building Blocks template library (TBB) employs a work stealing scheduler to balance computational loads across multiple cores. Benchmarks were performed on a workstation with two Xeon E5-2609 CPUs running at 2.5 GHZ, with 32 GB of ram, and on an attached Xeon Phi 7120P coprocessor with 61 cores running at 1.24 GHZ and 16 GB ram. Xeon Phi and Xeon CPU benchmarks were never run simultaneously.

## Experimental results

We evaluated pClay by measuring correctness in molecular solid generation, runtime performance, and accuracy of predictions made with pClay outputs. We measured the correctness of molecular solids in the “Accuracy of molecular solid generation” section, comparing outputs from pClay to molecular surfaces generated by established software. In the “Parallel Performance and Scaling” section, runtime performance was compared to VASP, an established software package that performs similar CSG operations. Parallel scaling was measured on both Xeon CPU and Xeon Phi hardware. Finally, we demonstrate the added prediction accuracy yielded by statistical models with data from pClay relative to VASP in the “Evaluating pClay on existing applications” section. Experimental datasets used for this work are detailed in Appendix B.

### Accuracy of molecular solid generation

While the generation of molecular surfaces is not the primary purpose of pClay, accurate comparisons require accurate molecular solids. To evaluate the molecular solids produced by pClay, we compared them to molecular surfaces generated with the trollbase library, which generates surfaces for several widely used software tools, including GRASP2 [25], MarkUs [48], and VASP [21]. The 100 sequentially diverse protein structures of Dataset A were used for this comparison.

First, we compared the volume within surfaces generated by pClay to the volume within surfaces generated by trollbase. Surfaces produced by pClay contained .00173% greater volume, on average, than those generated with trollbase. The largest volume difference was observed between surfaces generated for yeast RPN14 (pdb: 3VL1, chain A). That percentage difference was .02343%, and it arose from many small variations, accumulating to a total difference of $11.594\AA ^3$. The pClay surface contained $49,460\AA ^3$ and the trollbase surface contained $49,471\AA ^3$.

**Fig. 9:**
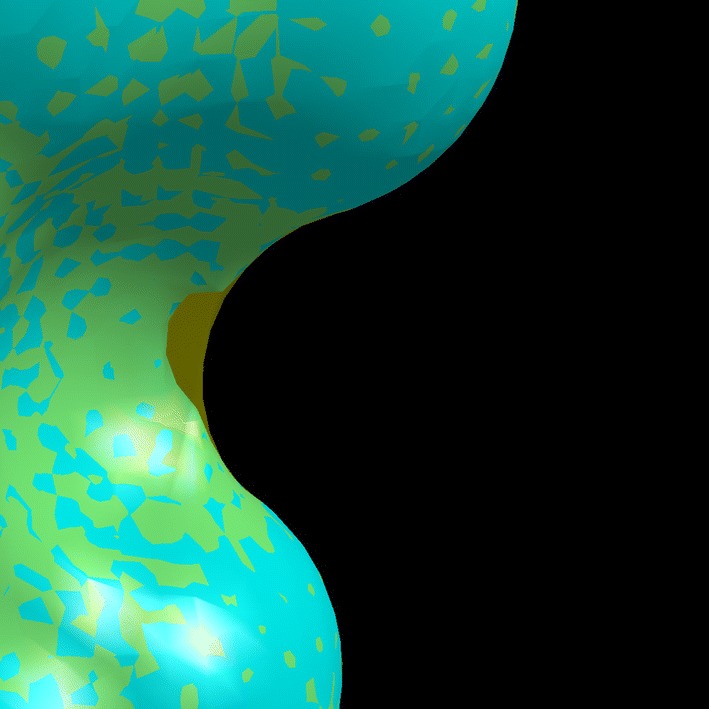
Close-in comparison of a molecular surface from pClay (teal) and trollbase (transparent yellow) generated from proto-oncogene C-FOS, (pdb: 2WT7). The notch where the surfaces are most distant yielded the largest displacement distance in Dataset A

Second, we measured displacement distances throughout surfaces generated with pClay and those of the same protein generated with the trollbase library. Molecular surfaces approximated from solids produced with pClay were polyhedral meshes with an average of 197,718.54 points. The average displacement distance over all surface points, averaged over all proteins, was 0.00383 Å. Average displacement distance varied only within a narrow range, having a standard deviation of 0.0004 Å. The smallest average displacement distance, observed on a UVB resistance protein (pdb: 4DNU, chain A) was 0.00315 Å and the largest average displacement distance, observed on a segment of an acetylcholine receptor (pdb: 1A11, chain A) was .00506 Å. Over the entire dataset, the average maximum displacement distance was .13619 Å. The largest maximum displacement distance in the entire dataset was .22024 Å, observed on proto-oncogene C-FOS (pdb: 2WT7, chain A, shown in Fig. 9). In this case and in others, the reason that maximum displacement on any protein can even rise to these modest levels stems from the fact that very thin spindles can occupy volume inside a lattice cube without occupying any corner of the cube, preventing it from being part of the triangular mesh output.

Overall, the volume within of molecular surfaces generated with pClay and trollbase are nearly identical and the distances between the two surfaces, evaluated at many points, are very close. These results demonstrate the pClay produces very accurate solids.

### Parallel performance and scaling

We evaluate the runtime performance of pClay by measuring the time required to perform two categories of CSG operations. We compared pClay performance to that of VASP on the same CSG operations. For both methods, runtimes included the time necessary to generate triangulated meshes of the output, in addition to the CSG operations themselves, even though pClay does not require it. We distinguish Xeon CPU cores from Xeon Phi cores by referring to them as CPU and PHI cores.

**Fig. 10:**
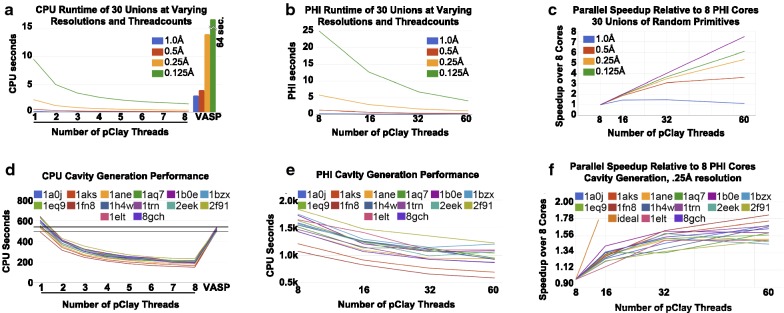
**a** Time to compute the union of 30 random primitives at varying resolutions and CPU cores. VASP performance (single threaded) is shown in vertical bars. **b** Time spent to compute the unions on PHI cores. **c** Parallel speedup on PHI cores. **d** Time spent for pClay to produce several binding cavities on CPU cores, compared to single-core VASP. **e** Time to produce the same cavities on PHI cores. **f** Parallel speedup of pClay in cavity production on varying PHI cores

The first category of CSG operations was a set of unions on the 30 randomly generated primitives of Dataset B, with mesh outputs to be generated at resolutions 1.0 Å, .5 Å, .25 Å and .125 Å. 30 primitives were selected to evaluate the performance of pClay in scenarios at least as challenging as in existing applications, which used approximately 20 primitives (e.g. [21]). CSG trees of these unions were balanced binary trees, but imbalanced trees yielded essentially identical runtimes. Since VASP does not use primitive representations, triangle meshes nearly identical to the primitives of Dataset B were provided as inputs to VASP. pClay and VASP runtime data, on between 1 and 8 CPU cores, are shown in Fig. 10a. On a single CPU core, pClay required .113 s to compute the CSG union on the 30 primitives of Dataset B at 1.0 Å resolution, whereas 9.492 s were required for a single core to compute the same union at .125 Å resolution. As the number of CPU cores increased to 8, runtime rapidly diminished to .03 s to compute the union at 1.0 Å resolution, and 1.465 s to at .125 Å. In contrast, single-threaded VASP required 3 s to compute union on 30 primitives at 1.0 Å resolution, and 64 s at .125 Å resolution. It is clear that pClay outperforms VASP, the current state of the art, on this union of geometric primitives.

We ran the same union operations on 8, 16, 32, and 60 Xeon Phi cores. Due to the slower speed of PHI cores relative to CPU cores, runtimes were slower even though the same number of computing threads were used in some cases. As the number of utilized cores increased, runtimes exhibited sublinear improvement (Fig. 10b), because communication overhead increases with the number of parallel threads. Runtimes for unions on coarser resolutions improved less than for finer resolutions. This difference in parallel speedup (Fig. 10c) arises from the fact that the problem size for coarser resolutions is already quite small and communications and setup time outweigh the advantages of parallelism. In contrast, finer resolutions create more computation to be divided, justifying the costs of communications and setup.

The second category of CSG operations followed the method illustrated in Fig. 1 to represent binding cavities as geometric solids. This method, developed and tested in earlier work [21], was applied to the serine proteases in Dataset C at .25A resolution. This resolution was selected because it is more detailed than resolutions used in existing work while remaining practical for comparison against VASP. Figure 10d illustrates the runtimes for generating the binding cavities. Cavity generation on a single CPU core completed in between 493 seconds and 643 seconds. Scaling up to 8 CPU cores, the same process required between 149 and 233 seconds. In comparison, single threaded VASP required between 499 and 538 seconds to perform the same work. While single threaded pClay was slower than VASP on one case, it became nearly two minutes faster in all cases by adding a second thread of computation, and faster still when adding more cores. Resources like this would be commonly available in most computers today.

The cavity generation experiment was rerun on 8, 16, 32, and 60 Xeon Phi cores. Again, since individual PHI cores are slower than individual CPU cores, runtimes for the same number of threads were slower on PHI cores. As the number of compute threads increased, we observed that runtimes fell subtly (Fig. 10e). Substantial increases in the number of PHI cores resulted in only modest improvements in runtimes (Fig. 10f). Runtimes on PHI cores contrasted from runtimes on CPU cores, where performance improved substantially with increases in the number of available cores. Since PHI performance scaled well on Dataset B, and since the molecular solids and spheres used to generate solid representations binding cavities are simply large collections of primitives, these results indicate that some aspects of the Xeon Phi architecture may be causing a bottleneck that does not exist in the case of CPU cores.

### Evaluating pClay on existing applications

The added precision of pClay enhances prediction accuracy in existing applications. To evaluate accuracy enhancement, we tested one such application by producing training data for VASP-S, a statistical model for detecting differences in ligand binding specificity with steric causes [16]. VASP-S is trained on the volumes of contiguous CSG differences, called *fragments*, that are computed from cavities with the same ligand binding preferences. This training enables VASP-S to estimate the probability (the *p*-value) that two cavities have similar binding preferences. If, for a given pair of cavities, *p* is lower than a threshold $\alpha$, VASP-S rejects the possibility that two cavities have similar binding preferences and predicts that they have different preferences. The VASP-S method is paraphrased in Appendix A.

**Fig. 11:**
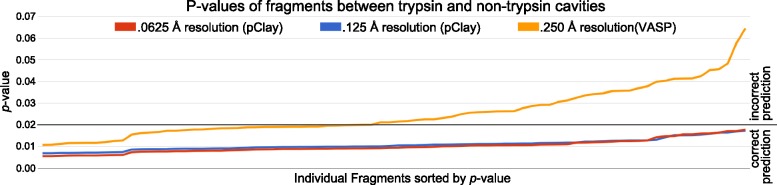
The *p*-value of the largest fragment between every trypsin-elastase and trypsin-chymotrypsin pair in Dataset C was estimated using training data generated at .0625 Å (blue line), .125 Å (red line), and .250 Å (yellow line). The vertical axis plots *p*-value, fragments are sorted by ascending *p*-value along the horizontal axis. The black line indicates the $\alpha$ threshold of 0.02. Fragments with *p*-values lower than 0.02 result in a rejection of the null hypothesis and thus a prediction that the elastase or chymotrypsin in the pair has binding preferences that are different from that of trypsin. *p*-values above the threshold do not result in a rejection of the null hypothesis and thus the incorrect presumption that an elastase or chymotrypsin has binding preferences similar to trypsin. The finer resolution training data made possible with pClay yielded more accurate predictions

We hypothesize that training the VASP-S model with data generated at finer resolutions, which is not possible without pClay, will produce more accurate predictions than a VASP-S model trained with coarser data. To evaluate this hypothesis, we used cavities from the trypsins in Dataset C, which all prefer to bind positively charged amino acids. Fragments were computed at the highest practical resolution for VASP, 0.25 Å, and at two new resolutions, 0.125 Å and 0.0625 Å, that are now possible with pClay. Fragments generated at each resolution were used as separate training sets for VASP-S, producing three separately trained versions of the VASP-S model that differ in the resolution of their training data. Next, we estimated the *p*-value of the largest CSG difference between every trypsin and every non-trypsin in Dataset C, using all versions of the VASP-S model (Fig. 11). Since the non-trypsins prefer ligands that are very different from those preferred by trypsin, we expect VASP-S to produce estimates of *p* that are below $\alpha$ (0.02).

When trained with data generated at the standard 0.25 Å resolution, VASP-S predicts that 43 of the 81 CSG differences between trypsin and non-trypsin cavities had different binding preferences. There were thus 38 false negative predictions where VASP-S incorrectly overlooked structural differences between cavities with different binding preferences. When trained with data generated at resolutions of 0.125 Å and 0.0625 Å, VASP-S made none of the same false negative prediction errors: This result is apparent in Fig. 11 where the yellow line, representing the model trained at 0.25 Å resolution intersects the $\alpha$ threshold of statistical significance near the center of the figure. In contrast, the red and blue lines, which plot *p*-values from models trained at 0.125 Å and 0.0625 Å, never cross the $\alpha$ threshold. Thus, statistical models trained with data from pClay had a 0% false negative rate. These results demonstrate that pClay can provide precision sufficient to ensure that statistical models do not lose accuracy from imprecisely generated training data.

## Conclusions

We have presented pClay, the first parallel algorithm for performing CSG analyses of protein structures and electrostatic isopotentials at arbitrarily high resolutions. Central to this capability is the use of mathematically exact primitives that can be assembled into molecular solids and parallel algorithms for computing CSG operations with multiple computing cores.

We have shown that the molecular solids produced with pClay are nearly identical to molecular surfaces generated by existing, widely used software. The volumes of molecular solids were shown to be close to those produced by an existing method within thousandths of one percent. When compared at nearly two hundred thousand positions, on average, surfaces produced with pClay differed from surfaces produced with an existing method by thousandths of an angstrom on average. Wheres the accuracy of earlier methods was important for productive visualization, these detailed validations, which, to our knowledge, have never been performed for existing methods, are more important for pClay because they ensure that pClay is making accurate comparisons on the molecular surfaces it generates.

We have also shown that pClay performs both artificial and practical CSG operations efficiently, and that performance scales with more processor cores. Our performance evaluation used both Xeon CPUs and a Xeon Phi coprocessor. We observed scalable performance on all tests, though performance scaled more modestly in the case of the Xeon Phi on cavity generation. These results show that parallelism can be used to drive both efficiency and precision, which can be crucial for applications that require a large amount of precise structural analysis.

The combination of parallelism and precision enables existing applications of CSG-based comparison to be enhanced with greater prediction accuracy. We demonstrated one such enhancement in our results, where we used data from pClay to train a statistical classifier to predict elements of protein structures that sterically cause differences in binding specificity. In comparison to training data produced with earlier methods, the training data produced with pClay was generated with superior geometric precision, leading to more accurate estimation of statistical significance. As a result, false negative predictions were eliminated from models using the more precise training data. By enhancing precision, pClay enables existing methods to avoid overlooking elements of protein structure that affect specificity.

These capabilities point to applications where steric or electrostatic influences on binding specificity need to be detected. As high throughput technologies increasingly reveal how disease proteins might vary between or within individuals, pClay offers the opportunity to examine that data and explain how specific elements of protein models could alter binding, thereby generating individualized insights into how drug therapies might be evaded, how molecular interactions might change, and how protein therapies can be redesigned for improved specificity.

## Figures and Tables

**Fig. 12:**
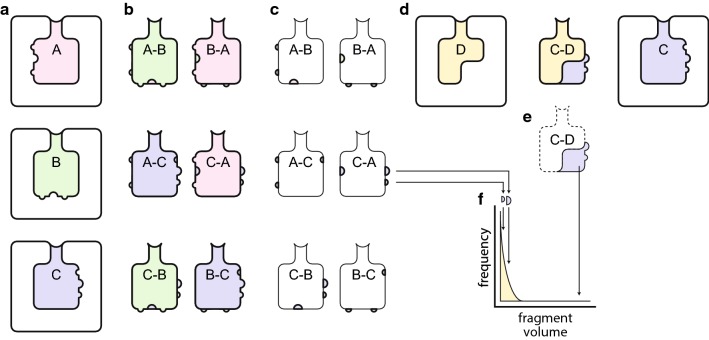
**a** Cavities *A*, *B* and *C* have the same binding preferences with subtle steric differences. **b** To produce training data, all symmetric CSG differences are computed between the training set cavities. **c** Individual fragments (semicircles) are separated from each CSG difference, and their volumes are computed. A log-normal probability density function is fitted to the volume data. **d** To estimate the probability that a given cavity *D* has similar or different binding preferences, a solid representation of the cavity is first generated (yellow) and symmetric CSG differences are computed between *D* and a randomly selected training set protein *C*. **e** The largest fragment found in the CSG differences $C-D$ and $D-C$, is shown in blue. **f** The log normal distribution enables the *p*-value to be estimated resulting in high probabilities for small fragments and low probabilities for large fragments

## Data Availability

All data is publicly available and reference in Appendix.

## Appendix A: VASP-S: a statistical model of steric variation in ligand binding cavities

When examining the molecular surfaces that define the geometry of binding cavities, small differences between cavity shapes correspond to variations in the pattern of steric hindrance in the two cavities. In two binding cavities, these steric variations can occur in several locations, where each location has individual potential to accommodate or hinder a potential ligand. We refer to these variations as *fragments*, and define each of them as a contiguous region of a CSG difference between two binding cavities. Where fragments are large (e.g. *C*–*D* in Fig. 12d), there are large variations in steric hindrance that may cause differences in binding specificity, and where fragments are small (e.g. *A*–*B* in Fig. 12b), the variations may be too subtle to hinder binding through a steric mechanism.

VASP-S is a statistical model of fragment volume. It is trained on the volumes of fragments from cavities that have the same binding preferences. It estimates the probability *p* that the volume of a given fragment could have arisen from cavities with binding preferences identical to those of the training set. If *p* is close to 1.0, then the probability is high that the fragment could have arisen from the training set, so we predict that the cavities that yielded the fragment exhibit binding preferences similar to those of cavities in the training set. If *p* is less than a threshold value $\alpha$, then the probability is so low as to suggest that the cavities that yielded the fragment are atypical of the training set. In such cases, we reject the null hypothesis that the given pair of cavities have identical binding preferences, and predict that they have different binding preferences. To paraphrase the VASP-S method [16], we will explain how VASP-S works in two stages: First, we document how VASP-S is trained, and then we explain how VASP-S is used to estimate *p*-values.

To train VASP-S, we begin with a collection of binding cavities with identical binding preferences (Fig. 12a). All cavities are aligned using the appropriate structure alignment software. Whole-structure alignment algorithms like ska [53] can be used for remotely homologous proteins, or cavity-based alignment software like MASH [4] can be used to align only cavities, when protein folds are totally dissimilar.[4]. This work performed alignments with ska. Next, a solid representation of every cavity is produced using the method described in the “solid representations of binding cavities” section. Then, for every pair of cavities *A*, *B* in the training set, the CSG differences *A*–*B* and *B*–*A* are computed (Fig. 12b), and we separate individual fragments from each CSG difference.

Figure 12c diagrams how fragments can result from CSG differences. For example, the CSG difference *A*–*B* results in three disconnected semicircular regions shown in red with heavy black outlines. These differences represent the many small variations that can occur between proteins with identical binding preferences. Even though they prefer the same ligand, substitutions in the amino acids adjacent to the cavity, differences in rotamer conformation or differences from protein breathing can create subtle differences that could be observed even between two crystal structures of the same protein. In practice there can be many dozens of such fragments, most of which are too small to have a steric influence on specificity. Using a graph method detailed in [17], we isolate individual fragments $f_i$ and record their volume $v(f_i)$. Once we have recorded all $v(f_i)$, we fit a log-normal distribution to these volumes to model the range of individual differences that could be observed between cavities with this binding specificity. We scale the height of the distribution so that the total area under the curve is equal to 1.

To make a prediction on the binding preferences of a given cavity *D*, we first select at random a cavity from the training set. In the example in Fig. 12, we select cavity *C*. We then generate a solid representation of the cavity in *D*, as before, and align the cavity to that of *C* in using the same alignment method. Next, we compute the CSG differences *C*–*D* and *D*–*C*, and identify the largest fragment *k* (Fig. 12e). *D*–*C* also produces small fragments, which are not shown in Fig. 12d for brevity. Next, we measure the area underneath the log-normal distribution to the right of *v*(*k*). This area represents the proportion of individual differences from the training set that had volume larger than *k*, and thus the probability *p* of observing an individual difference from the training set with volume equal to or larger than *v*(*k*). Note that even though cavity *C* is part of the training set it does not affect the independence of the fragments in *D*–*C* because *D* is completely independent of the training set; it arises from a different protein. If *p* is large, as in the case of the small semicircles (Fig. 12f), then *k* would be typical of the training set, so we would maintain the null hypothesis that cavity *D* has binding preferences similar to those of the training set. However, if *p* is smaller than a threshold of tolerable improbability $\alpha$, such as 2%, we would predict that cavity *D* is too atypical of the training set, and therefore that *D* has binding preferences that differ from the training set.

## Appendix B: Data sets used in this study

### Dataset A

Three data sets were used to benchmark and demonstrate the capabilities of pClay. To test the accuracy of molecular solids generated by pClay, Dataset A is a diverse selection of protein structures. This selection was made by using the VAST server [54] to produce sequentially nonredundant protein structures with a BLAST p-value cutoff of 10e−7. 100 proteins, listed in Table 1 were arbitrarily selected from this set.

**Table 1:** Dataset A: PDB codes of 100 sequentially non-redundant protein structures

1914A	1DX5I	1LKKA	1V6SA	2CSHA	2QHLC	3F6CA	4DGMA
19HCA	1EAYD	1LUGA	1WMDA	2DBAA	2TRCG	3F8VA	4DNUA
1A11A	1EFYA	1M2RA	1YPQB	2E3WA	2TRCP	3G46A	4DVCA
1A2OA	1FCYA	1MC2A	1ZHNA	2ED0A	2VB1A	3JXBC	4ESPA
1AF6A	1G2BA	1MUWA	1ZK4A	2ETZA	2W72B	3KLOA	4FAYA
1AG4A	1GCIA	1NA0A	1ZLMA	2GF5A	2WT7A	3KQNA	4FYYA
1AIEA	1GVPA	1OSDA	2A3VB	2J7ZA	2YUQA	3O79B	4G9SA
1AMMA	1HQSA	1P47A	2ADRA	2JXBA	3A10A	3QM9A	4GCNA
1AY7B	1I2KA	1PN9A	2ANVA	2K0XA	3D9AL	3U25A	4HVWA
1B0BA	1IT2A	1QHQA	2APFA	2NLLB	3DJ9A	3U8OL	
1CKAA	1JBEA	1R1PB	2BNUA	2OQ1A	3EG3A	3UBUB	
1CL7I	1K3YA	1R64A	2BZZA	2P49B	3ERXA	3VL1A	
1DEMA	1K5NB	1S9KE	2C4FU	2Q20B	3F00A	4AJ8A	

### Dataset B

To demonstrate the parallel scalability of pClay, Dataset B was composed of 10 sphere, 5 spindles, and 15 tetrahedron primitives. 30 primitives were selected to make the CSG operation at least as computationally complex as in earlier applications, which used 20 spheres [21]. The spheres were defined with a 5 Å radius and centered at atom coordinates. While the atoms overlapped very little, they were close enough that many overlaps existed between the spheres. To produce a scenario at least as complex, Dataset B contains randomly selected primitives of different types that are generated inside a $10\AA ^3$ cube.

**Table 2:** Spindles in dataset B

$c_x$	$c_y$	$c_z$	$p_x$	$p_y$	$p_z$	$r_maj$	$r_min$	endcap1	endcap2
2.9283	3.9283	6.2286	$-$ 0.6804	$-$ 0.6804	0.2725	4.9158	4.6742	$-$ 3.1616	2.3346
5.9856	6.1531	5.2775	0.7379	$-$ 0.5270	0.4216	4.0370	4.7434	$-$ 3.7995	3.3740
3.0000	4.0000	8.5000	$-$ 0.9863	0.0000	$-$ 0.1644	4.0206	3.0414	$-$ 1.8348	1.8348
4.8566	6.6324	5.7757	0.8137	0.4650	$-$ 0.3487	5.2327	5.3012	$-$ 2.9534	3.6970
4.0000	7.0000	6.5000	$-$ 0.5963	0.2981	0.7454	4.1733	4.3541	$-$ 2.7276	2.7276

Dataset B has 5 spindles. The torus describing the spindle is centered at $\{c_x, c_y, c_z\}$. The vector perpendicular to the plane of the torus is oriented in the direction of $\{p_x, p_y, p_z\}$. The distance between the torus center and the center of the probe is $r_maj$, and $r_min$ is the radius of the probe. Endcap1 and endcap2 indicate the distance of the endcaps of the spindle from the center along the perpendicular vector

The positions or corners of each primitive were randomly assigned integer values within specific ranges to ensure a large degree of overlap between primitives, as in [21]. In addition to reflecting existing applications, large degrees of overlap require the basic functions to be run up and down the CSG tree, thereby throughly testing pClay, whereas a scenario with few overlaps would be less challenging.

Spheres were generated with radius between 1 Å and 3.5 Å. Spindles were generated between spheres that were at least 5 Å apart, with radius between 1 Å and 2.5 Å, and a probe radius between 5 Å and 10 Å. These criteria generate both whole and broken spindles. Tetrahedra were generated with between 2.5 Å and 5.0 Å between two corners. These ranges created randomized primitives that inevitably overlapped with a convoluted overall shape.

These primitives are detailed in Tables 2 and 3.

**Table 3:** Tetrahedra and Spheres in Dataset B

Tetrahedra in dataset B												Spheres in dataset B			
$a_x$	$a_y*$	$a_z$	$b_x$	$b_y$	$b_z$	$c_x$	$c_y$	$c_z$	$d_x$	$d_y$	$d_z$	*x*	*y*	*z*	*r*
6	0	6	2	6	1	2	0	2	9	2	2	9	7	3	1
0	3	6	3	2	0	6	1	5	4	9	2	6	8	9	1
1	7	7	2	7	2	1	1	5	6	5	6	5	4	8	2
1	2	9	9	4	8	3	8	5	6	1	1	4	0	6	1
8	1	0	9	8	4	3	0	4	2	4	7	5	4	4	2
8	9	3	5	1	1	0	8	5	8	4	7	6	8	3	1
1	0	3	9	0	8	5	6	6	6	0	2	3	3	8	1
9	9	1	6	1	3	4	9	1	1	9	6	7	6	8	2
5	0	2	3	6	0	9	4	3	5	1	7	3	0	1	2
3	1	4	6	9	4	8	8	9	2	8	8	9	1	5	1
9	0	3	6	8	7	3	3	7	9	6	0				
1	0	4	8	6	2	4	7	9	3	9	2				
9	7	8	5	7	0	4	8	5	8	2	6				
1	3	7	0	1	0	7	2	6	4	5	2				
9	9	4	2	9	0	5	1	0	8	6	0				

The 15 tetrahedra in Dataset B have corners indicated by three dimensional coordinates at *a*, *b*, *c*, and *d*. The 10 spheres in Dataset B have centers at *x*, *y*, *z*, with radius *r*

### Dataset C

Finally, to demonstrate the accuracy of predictions made with pClay, Dataset C was created as a set of binding cavities from a sequentially nonredundant representative subset of the trypsins, chymotrypsins, and elastases. These three families of proteins were selected because they perform the same catalytic function, the hydrolysis of peptide bonds, and because each family prefers to hydrolyze peptide bonds following a different kind of amino acid. Trypsins prefer to hydrolyze peptide bonds after positively charged amino acids, chymotrypsins prefer to hydrolyze peptide bonds after large hydrophobic amino acids, and elastases prefer to hydrolyze peptide bonds after small hydrophobic amino acids. These distinctions represent a useful test set for evaluating the accuracy of pClay at distinguishing their active sites.

Beginning with 582 possible structures in the PDB, we eliminated one member of any pair of proteins with greater than 90% sequence identity, resulting in a selection of proteins with 47% average sequence identity. This process resulted in the structures specified in Table 4. Solid representations of the S1 cavity in each protein were generated using the method described in Fig. 1f.

**Table 4:** PDB Codes of dataset C structures

Chymotrypsins		Trypsins			
1EQ9	8GCH	1A0J	1AKS	1ANE	1AQ7
Elastases		1BZX	1FN8	1HRW	1TRN
1B0E	1ELT	2EEK	2F91		
